# Implementation Documentation and Process Assessment of the PharmNet Intervention: Observational Report

**DOI:** 10.2196/54077

**Published:** 2024-03-18

**Authors:** Lori Ann Eldridge, Beth E Meyerson, Jon Agley

**Affiliations:** 1 Department of Health Education and Promotion College of Health and Human Performance East Carolina University Greenville, NC United States; 2 Harm Reduction Research Lab, Family and Community Medicine College of Medicine-Tucson University of Arizona Tucson, AZ United States; 3 Comprehensive Center for Pain and Addiction University of Arizona Health Sciences University of Arizona Tucson, AZ United States; 4 Prevention Insights, Department of Applied Health Science School of Public Health Bloomington Indiana University Bloomington Bloomington, IN United States

**Keywords:** naloxone, Narcan, pharmacy, harm reduction, PharmNet, overdose, opioids, implementation, pragmatic trial

## Abstract

**Background:**

The number of overdose deaths in the United States involving opioids continues to exceed 100,000 per year. This has precipitated ongoing declarations of a public health emergency. Harm reduction approaches, such as promoting awareness of, ensuring access to, and fostering willingness to use naloxone to reverse opioid overdose, are a key component of a larger national strategy to address the crisis. In addition, overdose reversal with naloxone directly and immediately saves lives. Because of pharmacies’ ubiquity and pharmacists’ extensive clinical training, community pharmacies are well-positioned, in principle, to facilitate naloxone access and education.

**Objective:**

In 2022, a single-site pilot study of PharmNet, a community pharmacy intervention incorporating naloxone distribution, awareness building, and referral, showed promising outcomes for both naloxone and resource distribution in the community. As a next step, this study was intended to be a pilot randomized controlled trial of PharmNet in 7 pharmacies. However, due to circumstances outside of the study team’s control, data collection was unable to be fully completed as planned. In keeping with open research standards, we transparently report all available data from the study and discuss trial barriers and processes. We do so both to provide insights that may inform similar studies and to avoid the “file-drawer” (publication bias) problem, which can skew the aggregated scholarly literature through nonpublication of registered trial results or selective publication of findings affirming authors’ hypotheses.

**Methods:**

This paper reports an in-depth implementation study assessment, provides the available observational data, and discusses implementation considerations for similar studies in independent (eg, nonchain) community pharmacies.

**Results:**

Retrospective assessment of study outcomes and fidelity data provided for robust discussion around how resource differences in independent community pharmacies (vs well-resourced chain pharmacies), as well as high demands on staff, can affect intervention implementation, even when leadership is highly supportive.

**Conclusions:**

Community pharmacies, particularly independent community pharmacies, may require more support than anticipated to be successful when implementing a new intervention into practice, even if it might affect estimates of real-world effectiveness. Further implementation science research is needed specific to independent community pharmacies. All study elements are outlined in the International Registered Report Identifier (IRRID) PRR1-10.2196/42373. Although this paper reports results associated with that registration, results and conclusions should not be given the weight assigned to findings from a preregistered study.

**International Registered Report Identifier (IRRID):**

RR2-10.2196/42373

## Introduction

### Background

In the last decade, hundreds of thousands of lives have been lost in the United States due to fatal opioid-involved overdoses [1], and annual overdose death rates (computed as 12-month year-over-year incidence) have remained over 100,000 in recent reports from the Centers for Disease Control and Prevention [2]. Additionally, overdose deaths represent over one-third of all accidental deaths in the United States [3], with the majority involving illicitly manufactured fentanyl, a synthetic opioid [4]. In the state of Indiana, where this study was conducted, preliminary data indicate 2250 overdose deaths in 2022 [5], around 71% of which involved fentanyl or other synthetic opioids [5].

Naloxone, an opioid antagonist, works by blocking the effects of opioids and can allow persons who have overdosed to resume normal breathing [6]. It is a safe and effective way to rapidly reverse the effects of opioid overdose and can prevent death [6,7]. Research has shown that overdose deaths decline when overdose education and naloxone distribution (OEND) efforts are undertaken in communities [8], and that OEND programs are both an effective [9,10] and cost-effective [11] strategy to address overdose fatality.

In 2018, the US Surgeon General issued an official recommendation that all US citizens carry naloxone [12]. By 2021, most US states, including Indiana, had begun to address the overdose epidemic using combinations of policies that generally included “Good Samaritan” laws and standing orders for naloxone at pharmacies [13]. More recently, the declaration of an opioid public health emergency was renewed in September 2023 [14], approximately a year after the release of the US *National Drug Control Strategy*, which outlines goals that include the expansion of access to evidence-based harm reduction practices, including naloxone [15].

Beyond the impact in terms of lives saved, individuals who are revived with naloxone and who also have opioid use disorder (OUD) have an opportunity to engage with an “Opioid Cascade of Care” [16], such as through treatment initiation with medications for opioid use disorder (MOUD) in the emergency department [17]. For these benefits to accrue, however, naloxone must be easily accessible by those who can use it to reverse an opioid overdose. In the United States, nasal formulations of naloxone were recently approved for over-the-counter sale [18], following a unanimous vote by Food and Drug Administration advisors [19]. While this change might in principle increase the number of people carrying naloxone, data from Australia suggest that it may be a helpful but not sufficient step to increase access [20].

Even as additional means of obtaining naloxone are developed (eg, naloxone vending machines [21]), community pharmacies will likely remain an important component of supporting naloxone access and education. In some populations, patients interact with their pharmacists more often than with their primary care physicians [22], and pharmacists have been identified as some of the most accessible health care providers in the United States [23,24]. Studies show that community pharmacists generally acknowledge their role in assisting in harm reduction [25-27], and an Arizona study found that community pharmacists were typically comfortable providing consultation around and dispensing naloxone, even when they were uncomfortable with other harm reduction approaches such as syringe dispensing without a prescription [28]. As such, community pharmacists are theoretically well-positioned to improve harm reduction education and accessibility of related supplies. At the same time, recent studies also continue to suggest that naloxone remains unavailable for purchase in some community pharmacies and may be less likely to be available in independent pharmacies compared to chain pharmacies [29].

### The PharmNet Intervention

To better understand the dynamics and potential of community pharmacy harm reduction practice, we completed multiple preliminary studies [26,28,30-35]. Our work examined naloxone access, pharmacist comfort with harm reduction, pharmacy-based research approaches, and the “fit” of the pharmacy environment for harm reduction–related services. The studies were conducted from 2016 through 2022, and many of them engaged community and academic pharmacists as part of the research team.

By the end of the process, we had determined that our initial conceptualization of a “short” or “minimally demanding” intervention (eg, the first draft of PharmNet, a 10- to 15-minute screening and brief intervention-style intervention) would likely be perceived as useful but not necessarily feasible without a substantive added support system (eg, financial and personnel). Further, there was variability in the a priori intervention components that would be available at different pharmacy types. For example, a systematic review [36] in addition to our own earlier research in Indiana [33] and a recent study in North Carolina [29] identified independent pharmacies as being significantly less likely to stock or dispense naloxone compared to chain pharmacies (eg, ability to dispense naloxone, even at cost, could not be assumed). While independent pharmacies are, in theory, more likely to be able to participate in a small intervention implementation program (eg, they do not need to seek approval from a large bureaucracy associated with a national or international chain), they also lack the financial and systemic resources that are available to chain pharmacies, which can have implications for stocking and dispensing of products, as well as pharmacists’ availability to undertake additional work.

Thus, the PharmNet intervention was modified with the goal “to study procedures that have as minimal an impact as possible on pharmacy costs and operational functioning while maximally facilitating harm reduction from opioid overdose—in other words, to find an optimal intersection point of those concerns” [37]. The overall goals of the finalized PharmNet intervention are outlined in a previous paper, which reported the results of our initial single-site pilot study [37]. For clarity, we excerpt them here verbatim:

1. Building awareness of naloxone availability at the site among patients (eg, the use of yard signs and scrolling messages on television screens).
2. Supporting awareness among pharmacists and pharmacy technicians about proactively offering naloxone (eg, customized post-it notes).
3. Facilitating service provision by conducting *a priori* negotiations and establishing written agreements with local nonprofits to facilitate a pipeline of no-cost naloxone for the pharmacy.
4. Emphasizing bidirectional naloxone provision (eg, pharmacist- and pharmacy technician–initiated offers in addition to patient-initiated requests), and
5. Facilitating referral by providing a physical, durable, and curated list of community resources that can be used by pharmacists, handed to patients, and placed into pharmaceutical bags.

Interestingly, these goals are generally aligned with the intervention approaches described in a scoping review of opioid counseling and naloxone services published after our study concluded [38]. The review found that “all studies incorporated naloxone recommendations into their pharmacy-based opioid counseling and naloxone services,” and that most programs focused mostly on OEND [38]. Further, most of our awareness and communication strategies were similar to those in the review, though only one study in the review used outdoor yard signs, and none appear to have established pathways for pharmacies to receive no-cost naloxone [38].

Our first implementation of PharmNet was in a single independent pharmacy in the Midwestern United States. In that 3-month single-site pilot study, naloxone dispensing by any means (paid or no cost) nearly doubled, increasing by 96.48% (+9.33 doses per month); this included both distribution of no-cost doses (+6 doses per month) and an increase in the monthly rate of naloxone sold (+3.33 doses per month) compared to the 3 months before the intervention [37]. An average of 2.85 referral and resource cards (1/4 page in size) customized to the local area were distributed to patients each day. The initial pilot study also allowed us to further adjust the procedures based on feedback from community pharmacists [37]. The most substantive change was a drop from 3 sets of different post-it reminders to one, because only one type was regularly used by the pharmacy staff members in practice.

To better understand the results, and to begin assessing the intervention’s scalability, we then developed and published a study protocol for a pilot randomized controlled trial (RCT) of PharmNet [39]. Our stated goal was “to facilitate an improved understanding of whether it is reasonable to believe that the PharmNet intervention causes increased dispensing of naloxone ...” and we proposed to test the hypothesis that, “Monthly naloxone dispensing (combined sales and no-cost distribution) will be significantly increased in the pharmacies implementing PharmNet compared to those in the control arm” [39].

### This Report

This paper was originally planned to describe the results from a small RCT of PharmNet based on our protocol paper that also served as a registered report (International Registered Report Identifier: PRR1-10.2196/42373) [39]. However, several events and circumstances beyond the control of the study team (eg, a COVID-19 outbreak among pharmacists) resulted in substantial deviations from the proposed approach and outcome measures. The conceptual magnitude of these differences and the resultant effects on data and procedures mean that it would be inappropriate to attempt to assert that the information reported in this paper serves as answers to our original research questions.

At the same time, it is important for us to provide a complete description of the study and any resultant observations and lessons learned. In doing so, we avoid contributing to the “file drawer” (publication bias) problem, wherein studies that are perceived to be unsuccessful or that have nonsignificant results are never published to the detriment of the scientific enterprise and those affected by it [40-42]. Publication bias has been described as being conceptually similar to the practice of *p*-hacking [43]; it can skew the perception of a field of study in a particular direction through suppression (either deliberate or inadvertent) of results that do not align with the scientists’ goals. This can happen in at least 2 ways. First, results from registered trials may never be published at all (a recent study of RCTs in New Zealand found that 27% of results had not been published more than a decade after the study registration date) [44]. This can produce the mistaken impression that not much is known about a particular approach. Second, a “failed” study may be repeated with some minor modifications, and the resultant data reported as though it was the originally registered trial. This can lead to overestimation of the intervention’s likely outcomes as well as reducing the likelihood that procedural and implementation considerations—some of which may be important—are fully and transparently addressed.

Therefore, we share the results of this study in the form of an observational report that includes (1) the findings that we were able to collect, (2) the methods to collect that information, as well as (3) specific information related to deviations from planned data collection and analysis. Then, we provide a detailed written record of fidelity or process checks and the results of those checks. Finally, the implications of the external issues leading to these events, including discourse around the role that implementation fidelity checks may play in the conduct of intervention studies, and of the unique nature of independent pharmacies, are discussed.

## Methods

### Study Design

**Figure 1:**
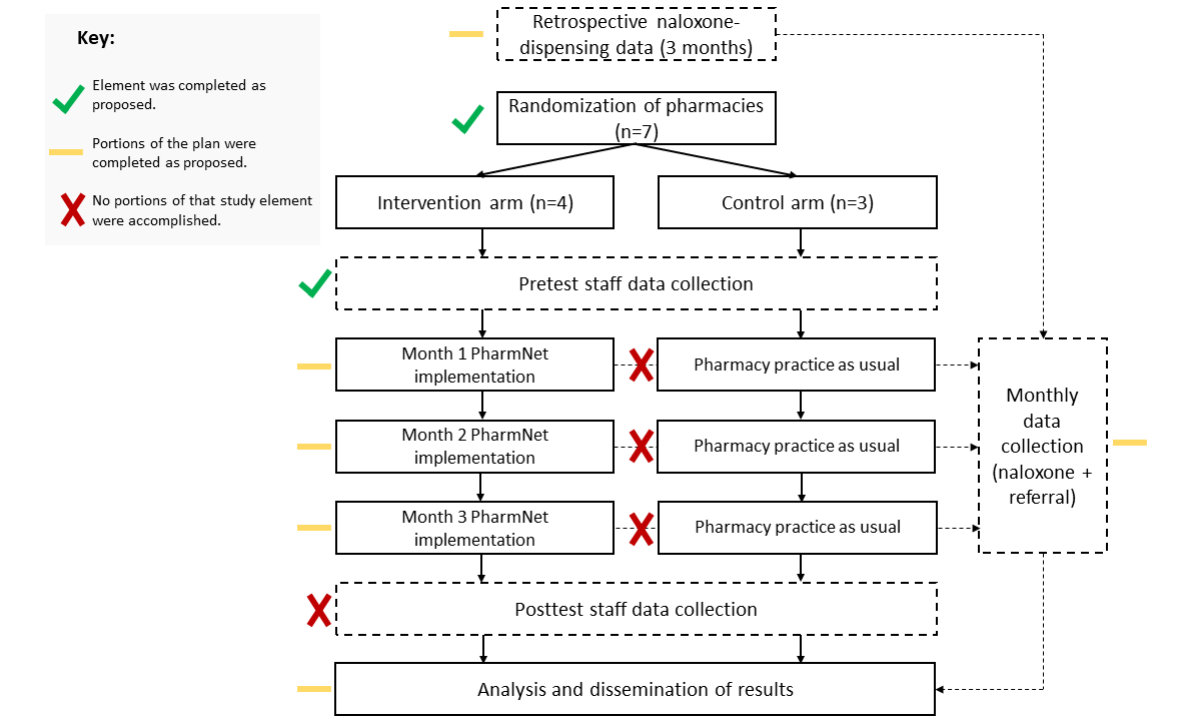
Original study protocol diagram for the PharmNet pilot trial, modified with indicators of completion status (adapted from Eldridge et al [39], which is published under the terms of the Creative Commons Attribution License 4.0 [45]).

We planned to conduct a pilot cluster RCT with 7 independent community pharmacies consisting of 2 parallel groups. In total, 4 pharmacies were allocated to the intervention arm, and 3 pharmacies were allocated to the control arm. The original project flow diagram is provided as Figure 1 [39,45], along with colored marks added after the study to indicate the degree of completion for that step.

Detailed implementation notes were collected from the 4 pharmacies randomly assigned to the intervention arm. We also obtained data on naloxone sales and no-cost distribution for the 3 months prior to the study period as well as for a period of 3 or more months after the intervention began (more details on this timeframe are provided subsequently). Some preintervention staff data are provided and discussed.

No data were collected from control pharmacies, no posttest data were able to be collected from pharmacy staff members, and no counts of referral cards were able to be obtained from any pharmacy.

### Sources of the Data

There were 2 types of participants in this study, *pharmacies* and *pharmacy staff members*.

- The 7 *participating pharmacies* were part of a single independent pharmacy organization. They were allocated simultaneously to a study arm using a web-based random sequence generator with a 4:3 ratio.
  These pharmacies all operate in the Midwestern United States and have a shared leadership structure (eg, there are individuals who oversee operations at all 7 pharmacies). In addition, each pharmacy has a managing pharmacist (which we call “lead pharmacists” in this paper to avoid conflating their management role with those of pharmacists overseeing the whole organization).
  Lead pharmacists manage day-to-day pharmacy operations at single sites.
- *Pharmacists and pharmacy staff members* at each of the 7 sites (both the intervention and control arms) were asked to participate in the study by providing individual-level data at pretest. Posttest data collection was planned but not completed (as described in the Results section).

### Intervention Description

The development of this intervention was described in detail in prior papers [37,39], and a structured table outlining the sequential steps of PharmNet was included in the published protocol paper [39]. The specific steps involved in the intervention are included in Tables 1 and 2, which describe each planned step (excerpted verbatim or paraphrased from the protocol paper, as appropriate, for clarity) as well as the actual outcome.

**Table 1:** Planned implementation steps and observed outcomes prior to the PharmNet study start date.

Intervention stage and planned steps		Outcomes
**Introduction to the study (prior to the start date)**		
	1. Digital video (.mp4) describing the intervention is distributed to the pharmacy manager.	An email was sent to the pharmacy organization management team, and they responded with an indication that they had received the video.
	2. Pharmacy manager disseminates the video.	The managing pharmacist reported they would show it to all 4 intervention pharmacies prior to the start date at their monthly employee meetings.
	3. All staff members view the video.	As far as we can determine, staff members viewed the video (based on phone conversations).
	4. Lead pharmacists are asked by the managing pharmacist to include the video in the orientation package for new pharmacists and pharmacy technicians who missed the initial meeting.	We were unable to verify whether this occurred.
**Early implementation (prior to the start date)**		
	1. Confirm with the managing pharmacist that all intervention pharmacies have viewed the video at the “all hands” staff meeting.	LAE spoke with the managing pharmacist on the phone on the start date to confirm this.
	2. Deliver the following supplies for each participating pharmacy: A set of 30 doses of naloxone (individually packaged Narcan nasal spray) to each pharmacy in numerically marked bags imprinted with “Not for Sale.” Deliver a set of 500 consecutively numbered harm reduction referral slips to each pharmacy (customized for each pharmacy location). Deliver sets of reminder post-it notes to pharmacies. Deliver the requested number of yard signs to each pharmacy.	JA verified all materials and personally delivered them to organizational management approximately 1 month prior to the start date. On the start date, LAE verified with the managing pharmacist that all 4 intervention pharmacies had received the supplies and that the intervention pharmacies were “live” with the intervention as of November 1, 2022.
	3. Ensure the pharmacy’s in-store television has access to the scrolling reminder text.	In principle, this was made available. As we note below, we cannot verify that it was used.

**Table 2:** Planned implementation steps and observed outcomes concurrent with the PharmNet study start date.

Intervention stage and planned steps		Outcomes
**Launch (on the start date)**		
	1. Verify the intervention has launched. Reminder post-its are placed in visible areas on the interior of the dispensing location (eg, staff side rather than patient side). Harm reduction referral cards are placed at each checkout for easy access. Yard signs are placed outside the building in visible locations where traffic (vehicle or foot) is common. Scrolling television banner will be enabled to run continuously through the intervention, reading “76.7% of overdoses happen at home. Bystanders who witness an overdose can be effective in reducing overdose mortality. Ask us about how you can save a life with naloxone today!”	Launch verification calls were made by LAE to the intervention pharmacies on November 2, 2022 (the day after launch). Pharmacy 1: The pharmacist who was working that day was a floating pharmacist and transferred LAE to the managing pharmacist. The managing pharmacist reported that the pharmacy “has the supplies and is ready to go.” Pharmacy 2: The pharmacist who was working that day said they had just returned from leave and did not know where the PharmNet intervention materials were in the pharmacy. They reported they would contact the managing pharmacist to find out where the materials were. The pharmacist stated they had watched the training video and were aware of the PharmNet intervention. Pharmacy 3: The pharmacist who was working that day reported they were not aware of receiving the PharmNet materials. They put LAE on hold to check if the supplies were in the pharmacy. They reported to LAE that they could not locate any supplies. Pharmacy 4: The pharmacist who was working that day reported that they had watched the training video and were aware of the PharmNet intervention. They reported that the pharmacy had not received any PharmNet supplies.
	2. Obtain additional details about the launch.	On day 4 of the intervention, LAE called the organizational management team. The primary purpose of the call was to request that they contact intervention pharmacies 2-4 to inform the pharmacists where the PharmNet materials were. LAE asked the managing pharmacist to inform the lead pharmacists at the intervention pharmacies to begin PharmNet if they had not already done so. The managing pharmacist informed the researcher that they would contact the pharmacists over the next few days. Unfortunately, soon after that call, we learned that the managing pharmacist had COVID-19 and was on sick leave. In addition, at least 1 additional lead pharmacist was also on sick leave for COVID-19, and pharmacists from other sites, and (when possible) the managing pharmacist, were often required to staff those roles when no additional staff members were available to do so.

### Data

The primary data points that we report include preimplementation naloxone sale data from intervention pharmacies (August 1, 2022, through October 31, 2022), postimplementation naloxone sale data from the same pharmacies (November 1, 2022, through January 31, 2023), and doses of no-cost naloxone distributed between the receipt of the doses at the end of May 2023. These data were obtained through objective pharmacy record extraction and physical (manual) counts of remaining naloxone doses by the lead pharmacist at each intervention pharmacy.

Data from individual staff members were collected via a survey administered prior to the implementation start date for PharmNet. The questionnaire was built in Qualtrics (Qualtrics), a secure web-based survey and data collection tool. The organizational pharmacy manager was provided with a link to distribute to pharmacists and pharmacy technicians at all independent community pharmacies in the study (both the intervention and control pharmacies). Measures included sociodemographic information, questions about pharmacy practice, comfort with harm reduction practices, and belief-based measures about people who use drugs. The wording for each item is provided in Multimedia Appendix 1. Completion of the survey was specifically not required for any individual pharmacist or staff member, but they were encouraged to participate. A copy of the full survey instrument is available as an appendix to the published protocol [39].

Finally, we also originally intended to have a single in-person fidelity check for each intervention pharmacy around 1 month into the project’s launch. For the reasons we describe in the Results section, multiple additional checks were added, and the modality was switched to telephonic and email correspondence to facilitate the increased frequency.

### Ethical Considerations

This study received ethics reviews prior to being initiated. Participation by pharmacies was designated as not human subjects research by the Indiana University institutional review board (document 12339). Participating pharmacy sites in the intervention arm were each offered an institutional incentive of US $1000 for their participation. Pharmacy employees completed a study information sheet prior to participating in the individual-level data collection and were compensated with a US $5 digital gift card for completing the pretest survey. Individual-level procedures were reviewed separately by the Indiana University institutional review board and received a designation of “exempt” (document 13956). Due to the small number of individual participants, data are reported only in aggregate and without cross-sectional tabulation.

## Results

### Pharmacy-Level Outcomes

On February 1, 2023, the intervention was concluded. From February through the first week of April 2023, we attempted to obtain pharmacy-level outcome data via phone calls, voicemails, in-person visits, and physical notes. However, these processes were unsuccessful. Our subjective observation is that a combination of illness and staffing complexity made it extremely difficult to collect the outcome data as originally planned in the protocol.

Although they were not originally involved in data collection, we were able to collaborate with the lead pharmacists at intervention pharmacies, who had participated in fidelity checking processes and were familiar with our team, to obtain the limited data set described in the Data section in May 2023. Importantly, we do not know the exact date when each pharmacy began offering no-cost naloxone doses, and the numbers of doses of no-cost naloxone dispensed were obtained several months after the intervention technically ended. As a result, we cannot compare naloxone distribution prior to and during the intervention period. However, we can still provide raw numbers (Table 3).

**Table 3:** Pharmacy-level naloxone sale and dispensing data, August 2022 through May 2023.

Pharmacy	Preimplementation naloxone sale data (August 1, 2022-October 31, 2022)	Implementation naloxone sale data (November 1, 2022-January 31, 2023)	Doses of no-cost naloxone dispensed (of 30) as of May 2023
Pharmacy 1	19	23	12
Pharmacy 2	45	19	30
Pharmacy 3	9	3	28
Pharmacy 4	11	10	24

### Pharmacy Staff Preintervention Data

A total of 8 staff members at least partly completed the study pretest during the period prior to the intervention, including 3 pharmacists and 5 pharmacy technicians. Pharmacy 4 was well-represented with 5 respondents, and there were 2 from pharmacy 3 and 1 from pharmacy 1. No staff members from pharmacy 2 or the control pharmacies participated.

All but 1 respondent who provided demographics (n=6) were female, and all were White, non-Hispanic, and heterosexual. Staff ages ranged from 32 to 52 years (n=5). Staff members who responded were comfortable with most harm reduction approaches, but fewer were comfortable with consulting with patients about pre-exposure prophylaxis for HIV prevention or dispensing syringes for nonprescription drug use. No participants indicated they were uncomfortable dispensing naloxone for overdose reversal or making referrals to community services. Further, staff member attitudes toward patients who use drugs or have OUD were not typically stigmatizing, though they expected that others (“most people”) might blame persons with OUD or believe that they are dangerous. A table of these data is provided in Multimedia Appendix 1.

### Fidelity Data

We also collected a full written accounting of the 9 distinct fidelity checkpoints during the intervention period. This information helped to contextualize some of the implementation issues around which we provide interpretation and discussion in the next section. Although not all study processes were in place at the time of the final fidelity check at week 10 of the 12-week intervention, by the time we collected outcome data, the intervention was apparently functioning at all sites (even though the technical “intervention period” was over). All documentation is available in Tables 4 and 5.

**Table 4:** Implementation fidelity checkpoint documentation for the PharmNet study through day 22.

Timeframe	Outcome
Day 10—Calls to all intervention pharmacies	Pharmacy 1: The pharmacist who answered the phone reported that they were not the usual pharmacist and only work 1 day a week, but that the pharmacy did have the yard signs and the no-cost naloxone. However, they reported that the yard signs were not placed outside the store. They also indicated that the managing pharmacist was on leave and that they recommend calling when they returned the following week to follow-up about the yard signs. Pharmacy 2: The pharmacist reported they were not the usual pharmacist and only work at the pharmacy 2 times a month, and that the regular pharmacist who works at that store would be back the following week. They did not know about PharmNet nor about the no-cost naloxone, yard signs, or harm reduction referral sheets. Pharmacy 3: LAE spoke with the same pharmacist as previously (the startup verification). The pharmacist reported that they did not think they had received PharmNet supplies, but that they were ready to start the intervention as soon as they received the supplies. Pharmacy 4: We spoke with the same pharmacist as previously (the startup verification). The pharmacist reported that they did not think they had received PharmNet supplies.
Day 14—Attempt to coordinate with the pharmacies	LAE attempted to reach the managing pharmacist at 2 separate pharmacies. A pharmacy technician called an additional 5 pharmacies but was unable to locate them. We left a voicemail at their office. We later learned that they were on leave during this period.
Days 16, 17, and 21—Follow-up calls, after which we learned about illnesses or leaves among the pharmacists	We called the original pilot pharmacy attempting to contact the leadership team. LAE left a voicemail and sent them an email on each of the 3 days.
Day 22—Calls to all intervention pharmacies	LAE learned that the managing pharmacist had returned to the office, but they were in a meeting when we called. A pharmacy technician took a message. Calls were also made to each of the pharmacies in the intervention arm. Pharmacy 1: When LAE called and requested to speak to a pharmacist, the pharmacy technician transferred her to the original pilot pharmacy location (which was not part of the study). LAE spoke with a pharmacist at this location, and this pharmacist reported they were unaware of the status of PharmNet at pharmacy 1 as they never worked at that site. They transferred the researcher back to pharmacy 1, where the pharmacy technician forwarded the researcher to voicemail. LAE left a message. Pharmacy 2: We spoke with a pharmacist who confirmed that the supplies had been received and that the intervention had begun. Pharmacy 3: The pharmacist reported that they still were not aware of having received the PharmNet supplies. Pharmacy 4: The pharmacist reported that they still had not received the PharmNet supplies. The pharmacist reported they had reached out to their leadership about the supplies and were informed that their supplies were located at pharmacy 1 and would be delivered after Thanksgiving.

**Table 5:** Implementation fidelity checkpoint documentation for the PharmNet study, week 4 through week 10.

Timeframe	Outcome
Remainder of week 4—Additional verification calls to intervention pharmacies to determine status and if any additional supplies were needed	LAE made calls to each of the pharmacies in the intervention arm (1) to determine if pharmacy 1 and 2 needed supplies and were following PharmNet protocols and (2) to assess if supplies were received and that PharmNet started at pharmacy 3 and 4. Pharmacy 1: We spoke to an on-call pharmacist who confirmed that they were following PharmNet’s protocol and that they had enough supplies (eg, harm reduction referral cards and no-cost naloxone). Pharmacy 2: The pharmacist confirmed that they were following PharmNet’s protocol and that they had enough supplies (eg, harm reduction referral cards and naloxone). Pharmacy 3: The pharmacist reported that they still were not aware of having received the PharmNet supplies. Pharmacy 4: The pharmacist reported that they still were not aware of having received the PharmNet supplies.
Week 6—Additional verification calls to intervention pharmacies to determine status and if any additional supplies were needed	LAE made calls to each of the pharmacies in the intervention arm (1) to determine if pharmacy 1 and 2 needed supplies and were following PharmNet protocols and (2) to assess if supplies were received and that PharmNet started at pharmacy 3 and 4. Pharmacy 1: The pharmacist confirmed that they were following PharmNet’s protocol and that they had enough supplies (eg, harm reduction referral cards and naloxone). Pharmacy 2: The pharmacist confirmed that they were following PharmNet’s protocol and that they had enough supplies (eg, harm reduction referral cards and naloxone). Pharmacy 3: The pharmacist reported that they still were not aware of having received the PharmNet supplies. Pharmacy 4: The pharmacist reported that they had not received all of the PharmNet supplies, but they had received the referral cards in the mail.
Week 10—Additional verification calls to intervention pharmacies to determine status and if any additional supplies were needed	LAE made calls to each of the pharmacies in the intervention arm (1) to determine if pharmacy 1 and 2 needed supplies and were following PharmNet protocols, (2) to assess if supplies were received and if PharmNet had started at pharmacy 3, and (3) to check if pharmacy 4 received the remaining supplies and started the intervention. Pharmacy 1: The pharmacist confirmed that they were following PharmNet’s protocol and that they had enough supplies (eg, harm reduction referral cards and naloxone). Pharmacy 2: The pharmacist confirmed that they were following PharmNet’s and that they had enough supplies (eg, harm reduction referral cards and naloxone). Pharmacy 3: There was no pharmacist available. We left a message requesting a callback, but it was never received. Pharmacy 4: The pharmacist reported that they had not received all of the PharmNet supplies, but that they were currently using the referral cards.

## Discussion

### Context of the Report

PharmNet was developed with independent community pharmacies in mind as a core implementation venue. This was partly because of trends around naloxone stocking and dispensing, as described in the Background section, which suggested that independent pharmacies might especially benefit from intervention support. In addition, there is a large segment of the US population, typically older, rural, and with lower income levels, for whom independent pharmacies are the primary point of pharmacy access (recent estimates place the number of individuals at over 15 million) [46]. Thus, it is particularly important to know whether interventions can operate effectively in independent pharmacy venues.

As we have previously indicated, our single-site pilot, which preceded this study, was successful [37], whereas this study encountered multiple barriers, even though they were conducted within the same small, independent pharmacy group. Our subjective qualitative assessment is that these were meta-structural barriers—perhaps common to independent pharmacies or related to the implementation decisions—and not a function of the individuals in the independent pharmacy group. Further, many of the barriers we encountered did not appear to be especially related to the program’s focal area (ie, naloxone distribution).

### Barriers Specific to Pharmacy Naloxone Distribution

The research literature has identified several barriers that can occur when implementing pharmacy-based OEND programs, but interestingly, this study of the PharmNet intervention never encountered most of these barriers (though it encountered others). In some cases, this may have been due to the intervention’s design, as we spent considerable time in the development phase determining how we might obviate frequently discussed implementation issues. For example, multiple publications have identified that reimbursement for products and services can be an implementation barrier (eg, for the costs of naloxone, pharmacist time, or other program features) [38,47]. In certain program structures (eg, where free or reduced cost naloxone doses are unavailable), patients’ out-of-pocket costs to purchase naloxone can also be a concern [48]. However, PharmNet included a planning step where the research team negotiated with nonprofit organizations to procure sources of no-cost naloxone. This meant that pharmacies could continue selling naloxone where appropriate, but that they also had the ability to provide free doses when indicated (eg, to patients who could not afford it) without needing to find a source of reimbursement.

Similarly, while pharmacy-based counseling is sometimes included in pharmacy OEND interventions, we were concerned about its feasibility in low-resource pharmacies, especially after COVID-19. Thus, for PharmNet, we opted to provide referral and resource cards in prescription bags that were tailored to each unique geographic location. In doing so, we attempted to avoid common barriers reported by similar studies such as lack of time or space for counseling [48]. Another larger study in 2 pharmacy chains also reported adaptations resulting from COVID-19, such as “streamlined counseling and drive-thru provision adaptations ...” [49].

Further, while a study of opioid overdose prevention programs in pharmacies identified barriers related to pharmacy leadership support [48], our perception is that the leadership team and pharmacy management in place at this independent pharmacy group were incredibly motivated, competent, and willing to support this intervention to the best of their ability—and it appeared that they were primarily limited by factors of raw capacity. In addition, though our pharmacist and pharmacy technician data were not collected from all intervention pharmacies, the limited data set that we procured did not identify any substantive concerns around comfort with dispensing naloxone.

### Barriers Related to Independent Pharmacies as Scalable Intervention Sites

We intentionally designed this pilot trial to be pragmatic, meaning that the implementation environment would mirror real-world practice [39]. Our primary concern was that the resources provided as part of an intensive trial support system would introduce a kind of heterogeneity of treatment effect [50], where pharmacies working to conduct the intervention outside of the context of the RCT would be systematically different by virtue of not having the same startup support. This would potentially inflate the effect size of the intervention within the study relative to its true effectiveness. Therefore, for individuals working in single pharmacies (eg, lead pharmacists, pharmacy technicians, and other staff members), we did not plan any direct face-to-face interaction with our team. Instead, the intervention materials themselves—introductory videos, instructions to the overall leadership of the organization, and materials, along with a planned fidelity check—were the primary means of dissemination.

However, the pharmacy practice environment is also experiencing a variety of substantive external pressures. For example, a 2022 survey from the National Community Pharmacists Association found nearly one-quarter of responding pharmacy owners or managers had difficulty filling pharmacist roles, and 88.8% had similar difficulty filling pharmacy technician positions [51]. A 2019 survey of full-time pharmacists found that even though independent pharmacists had higher job satisfaction, organizational commitment, and perceived control in the work environment than chain, supermarket, or mass-merchandiser pharmacies, those numbers had still declined over the past 5 years [52]. Strains on the broader pharmacy working environment have recently appeared in media reports as well [53]. Understanding these pressures and accounting for them with pragmatic intervention design will likely be important to sustainable implementation success.

We suspect that these or similar external stressors played a meaningful role in the outcomes of this trial. Lessons from remote decentralized clinical trials, for example, highlight how reducing the research burden on some elements of a team or partnership may inadvertently shift the burden onto others working in the field [54]. In other words, eventually, someone must do the work, and when staffing levels are low and exogenous stressors (eg, a COVID-19 outbreak) occur, an unreasonably large volume of responsibility may accrue to a small number of people. This is a particular concern due to the prevalence of pharmacist burnout, which a systematic review recently estimated to exceed 50% [55], though a slightly older review reported a lower mean percentage [56] (both reviews indicated heterogeneity among included studies). In this case, while we do not have (nor are intending to imply) any direct evidence of burnout, per se, we noted that there was not a “deep bench” of staff and that when illnesses or similar issues occurred in these independent pharmacies, the work was often covered by others who already had full-time (or greater) workloads.

In contrast, in one well-conducted, harm reduction–focused, pragmatic RCT of 175 community chain pharmacies, the researchers encountered both COVID-19 and wildfires [57]. Their study team conducted the majority of their fidelity checks face-to-face and had regular phone contact with the pharmacies. Their published protocol states the research team maintained a “close collaboration and regular meetings with the pharmacy corporate leadership.” Unexpected circumstances that arose were sometimes able to be mitigated by pharmacy corporate leadership (eg, the provision of additional pharmacists to relieve pharmacists from their regular work duties so that the onsite “academic detailing” could be conducted). These types of resources and supports may not be accessible to most independent pharmacies.

### Do Fidelity Checks Contribute to Intervention Effectiveness?

The major differences between our single-site pilot study (which was completed successfully and which produced positive outcome data) [37] and this follow-up study remain of interest to our team. One of our working theories relates to the nature of implementation fidelity checking that occurred. Implementation fidelity checking has long been understood as an important component of implementation science [58], though community pharmacy intervention studies do not always report implementation fidelity tools or processes [59].

Our single-site pilot study incorporated face-to-face fidelity checks on a regular basis [37]. This involved a study investigator regularly visiting the pharmacy in person, speaking with staff members and pharmacists, obtaining interim information, and identifying necessary points of action. In designing this follow-up study, we did not consider the effects that regular in-person fidelity checking might have had on intervention effectiveness in our single-site pilot. In other words, we do not know how much of the implementation effectiveness in the original study was a direct or indirect result of the routine physical presence of a researcher in the pharmacy. It is not necessarily the case that a single-site pilot is an insufficient first step to prepare for a larger-scale study, but rather, it seems that researchers might benefit from carefully examining the ways in which intensive fidelity procedures may actively contribute to successful implementation rather than merely documenting what happened.

### Limitations

As noted throughout this paper, there were numerous limitations that resulted in our reporting of fidelity encounter data and limited outcome measures. Even though this study was preregistered, due to the differences in the data collected from what was planned, we could not test the planned hypotheses. This study should therefore be considered exploratory. In addition, while we carefully consider the context of the study in our Discussion section, the ideas presented therein are hypotheses only and should not be interpreted as definitive statements of fact.

### Conclusions

Independent pharmacies, especially in rural areas, will likely continue to be key parts of US health care teams. For example, in April 2023, the Express Scripts business of Evernorth, a subsidiary of The Cigna Group, announced that they are extending their efforts to expand health care in rural communities via independent pharmacies [60]. As the prevalence of fatal opioid overdose remains high, multiple avenues to prevent fatality are warranted. Even though nasal formulations of naloxone are now available over the counter, pharmacies have a role in creating awareness and fostering accessibility for patients for whom the cost is prohibitive. As one peer reviewer (Steven H Linder MD) aptly noted, “the optimal means of naloxone community distribution remains to be determined.”

In terms of broader program implementation considerations for pharmacies, it may be the case—especially for independent pharmacies—that the start of a new initiative needs to be accompanied by more substantive support in the current pharmacy practice environment. If a trial is conducted over a longer period, support could in theory be withdrawn, and effectiveness measured longitudinally. However, we could not, with the data obtained from this study, disentangle issues around fidelity checking and externalities from those related to the overall pharmacist workload and burden and the intervention mechanics.

The strength of this paper is its detailed process description and transparent disclosure of the issues that affected the intervention. Given the likely importance of independent pharmacies to the national overdose response effort, we encourage further implementation science research that is specific to independent community pharmacies, which are often collapsed with other types of pharmacies but that have unique facilitators and barriers to intervention uptake.

## Appendix

Multimedia Appendix 1 Staff-level preintervention data.

## Abbreviations

MOUD: medications for opioid use disorder
OEND: opioid education and naloxone distribution
OUD: opioid use disorder
RCT: randomized controlled trial

## Data Availability

All data generated or analyzed during this study are included in this published paper (Multimedia Appendix 1), except for the raw data for the small sample (N=8) of staff members who completed the pretest, which are reported in aggregate but not at the individual level due to privacy concerns.
