# Correction: Implementation Documentation and Process Assessment of the PharmNet Intervention: Observational Report

**DOI:** 10.2196/59427

**Published:** 2024-04-11

**Authors:** Lori Ann Eldridge, Beth E Meyerson, Jon Agley

**Affiliations:** 1 Department of Health Education and Promotion College of Health and Human Performance East Carolina University Greenville, NC United States; 2 Harm Reduction Research Lab, Family and Community Medicine College of Medicine-Tucson University of Arizona Tucson, AZ United States; 3 Comprehensive Center for Pain and Addiction University of Arizona Health Sciences University of Arizona Tucson, AZ United States; 4 Prevention Insights, Department of Applied Health Science School of Public Health Bloomington Indiana University Bloomington Bloomington, IN United States

In “Implementation Documentation and Process Assessment of the PharmNet Intervention: Observational Report” (JMIR Form Res 2024;8:e54077) two corrections were made.

The peer reviewer information has been updated. The first line in the publication information box has been changed from:

...peer-reviewed by D Carpenter...

to:

...peer-reviewed by SH Linder, D Carpenter...

Additionally, the following sentence appeared within the “Conclusions” section:

As one peer reviewer aptly noted, “the optimal means of naloxone community distribution remains to be determined.”

This has been changed to:

As one peer reviewer (Steven H Linder MD) aptly noted, “the optimal means of naloxone community distribution remains to be determined.”

The correction will appear in the online version of the paper on the JMIR Publications website on April 11, 2024, together with the publication of this correction notice. Because this was made after submission to PubMed, PubMed Central, and other full-text repositories, the corrected article has also been resubmitted to those repositories.

