# Supplementary material for: Implementation Documentation and Process Assessment of the PharmNet Intervention: Observational Report
Source: JMIR Form Res. 2024 Mar 18;8:e54077. doi: 10.2196/54077 (PMC10985598; doi:10.2196/54077)
Supplement: Multimedia Appendix 1 [file formative_v8i1e54077_app1.docx]

| **I am comfortable…** | **n (%) Agree** | **n (%) No Opinion** | **n (%) Disagree** |
| --- | --- | --- | --- |
| …consulting with patients about safe syringe use. | 6 (85.7%) | 1 (14.3%) | 0 (0%) |
| …consulting with patients about safe syringe disposal. | 7 (100%) | 0 (0%) | 0 (0%) |
| …consulting with patients about safer sex practices. | 4 (57.1%) | 2 (28.6%) | 1 (14.3%) |
| …consulting with patients about the need for naloxone. | 6 (85.7%) | 0 (0%) | 1 (14.3%) |
| …consulting with patients about their need to reduce opioid misuse. | 6 (85.7%) | 1 (14.3%) | 0 (0%) |
| …consulting with patients about PrEP for HIV prevention. | 3 (50.0%) | 2 (33.3%) | 1 (16.7%) |
| …dispensing syringes for non-prescription injection drug use. | 2 (33.3%) | 1 (16.7%) | 3 (50.0%) |
| …dispensing naloxone for overdose reversal. | 5 (83.3%) | 1 (16.7%) | 0 (0%) |
| …dispensing PrEP for HIV prevention. | 4 (66.7%) | 2 (33.3%) | 0 (0%) |
| …making referrals to community services. | 6 (100%) | 0 (0%) | 0 (0%) |
| **Please indicate your level of agreement with the following statements:** | **Mean [1=Strongly Agree, 5= Strongly Disagree]** | | **SD** |
| I prefer not to work with patients who use drugs. | 4.50 | | 0.84 |
| I feel especially compassionate toward patients with opioid use disorder. | 1.83 | | 0.98 |
| Treating patients with opioid use disorder is a waste of medical dollars. | 4.67 | | 0.52 |
| I sometimes feel judged by my peers for seeking to serve patients with opioid use disorder. | 3.17 | | 0.75 |
| I don’t want my pharmacy to be known as a supporter of drug users. | 4.17 | | 0.75 |
| Most people believe that a person addicted to opioids is dangerous. | 2.00 | | 0.00 |
| Most people believe that a person who is addicted to opioids is to blame for their problems. | 2.00 | | 0.89 |
| I believe that a person who is addicted to opioids cannot be trusted. | 2.83 | | 1.17 |
| I think that a person who is addicted to opioids is to blame for their problems. | 4.17 | | 0.75 |
| I believe that patients who are addicted to opioids might cause trouble in my pharmacy. | 3.17 | | 0.75 |
